# Kuantim mi tu (“Count me too”): Using Multiple Methods to Estimate the Number of Female Sex Workers, Men Who Have Sex With Men, and Transgender Women in Papua New Guinea in 2016 and 2017

**DOI:** 10.2196/11285

**Published:** 2019-03-21

**Authors:** Damian Weikum, Angela Kelly-Hanku, Parker Hou, Martha Kupul, Angelyne Amos-Kuma, Steven G Badman, Nick Dala, Kelsey C Coy, John M Kaldor, Andrew J Vallely, Avi J Hakim

**Affiliations:** 1 US Centers for Disease Control and Prevention Atlanta, GA United States; 2 Kirby Institute UNSW Sydney Australia; 3 Papua New Guinea Institute of Medical Research Goroka Papua New Guinea; 4 Papua New Guinea National Department of Health Port Moresby Papua New Guinea

**Keywords:** Papua New Guinea, population size estimation, female sex workers, men who have sex with men, transgender women, key populations, respondent-driven sampling, unique object multiplier, service multiplier, successive sampling

## Abstract

**Background:**

Female sex workers (FSW), men who have sex with men (MSM), and transgender women (TGW) are at high risk of acquiring HIV in many settings, such as Papua New Guinea (PNG). An understanding of the approximate size of these populations can inform resource allocation for HIV services for FSW, MSM, and TGW.

**Objective:**

An objective of this multi-site survey was to conduct updated population size estimations (PSE) of FSW and MSM/TGW.

**Methods:**

Respondent-driven sampling (RDS) biobehavioral surveys of FSW and MSM/TGW were conducted in 3 major cities—(1) Port Moresby, (2) Lae, and (3) Mount Hagen—between June 2016 and December 2017. Eligibility criteria for FSW included: (1) ≥12 years of age, (2) born female, (3) could speak English or Tok Pisin (PNG Pidgin), and (4) had sold or exchanged sex with a man in the past six months. Eligibility for MSM/TGW included: (1) ≥12 years of age, (2) born male, (3) could speak English, or Tok Pisin, and (4) had engaged in oral or anal sex with another person born male in the past six months. PSE methods included unique object multiplier, service multiplier, and successive sampling-population size estimation (SS-PSE) using imputed visibility. Weighted data analyses were conducted using RDS-Analyst and Microsoft Excel.

**Results:**

Sample sizes for FSW and MSM/TGW in Port Moresby, Lae, and Mount Hagen included: (1) 673 and 400, (2) 709 and 352, and (3) 709 and 111 respectively. Keychains were used for the unique object multiplier method and were distributed 1 week before the start of each RDS survey. HIV service testing data were only available in Port Moresby and Mount Hagen and SS-PSE estimates were calculated for all cities. Due to limited service provider data and uncertain prior size estimation knowledge, unique object multiplier weighted estimations were chosen for estimates. In Port Moresby, we estimate that there are 16,053 (95% CI 8232-23,874) FSW and 7487 (95% CI 3975-11,000) MSM/TGW, approximately 9.5% and 3.8% of the female and male populations respectively. In Lae, we estimate that there are 6105 (95% CI 4459-7752) FSW and 4669 (95% CI 3068-6271) MSM/TGW, approximately 14.4% and 10.1% of the female and male populations respectively. In Mount Hagen, we estimate that there are 2646 (95% CI 1655-3638) FSW and 1095 (95% CI 913-1151) MSM/TGW using service multiplier and successive sampling, respectively. This is approximately 17.1% and 6.3% of the female and male populations respectively.

**Conclusions:**

As the HIV epidemic in PNG rapidly evolves among key populations, PSE should be repeated to produce current estimates for timely comparison and future trend analysis.

## Introduction

HIV disproportionally affects marginalized and stigmatized populations [1]. Female sex workers (FSW), men who have sex with men (MSM), and transgender women (TGW) are 3 key populations (KP) at greater risk for HIV [1-3]. Globally, FSW, MSM, and TGW are estimated to be 13.5 [2], 19.3 [3], and 48.8 [4] times more likely to be infected with HIV than the general population, respectively. This risk is accentuated by critical barriers to HIV-related prevention and treatment services, such as violence, criminalization, stigma, and discrimination [5-7].

Papua New Guinea (PNG) has the largest HIV epidemic in the Pacific region, with a national prevalence estimated at 0.9% [8]. Recent surveys of FSW and MSM/TGW estimated HIV prevalence among FSW and MSM/TGW at 14.9% and 8.5%, respectively, in the capital of Port Moresby. In Lae, the country’s economic hub, it was estimated at 12.9% and 7.1%, respectively [9].

Population size estimates can inform resource targeting and program monitoring [1]. The only population size estimation of FSW and MSM in Port Moresby using empirical methods was conducted in 2006 and utilized the service multiplier in conjunction with a respondent-driven sampling (RDS) survey [10]. Social changes such as population growth and increased mobility have occurred since then [11,12]. Updated size estimates are needed in Port Moresby and other cities, such as Lae and Mount Hagen, require first-time estimates. We used unique object multiplier, service multiplier, and successive sampling-population size estimation methods, which are direct and empirical, to estimate the number of FSW and MSM/TGW in these 3 cities.

## Methods

### Community Consultation

Community consultation was undertaken within each city. Population members recognized that MSM and TGW are distinct populations but TGW are too few to achieve adequate sample size as an independent RDS sample. The 2 populations thus agreed to be combined into a single sample.

### Recruitment

Seeds were purposively selected to be diverse with respect to age, sexual and gender identity, residence, region of origin, marital status, receipt of a unique object, and affiliation with a non-governmental or community-based organization.

### Data Collection

Separate RDS biobehavioral surveys (BBS) of FSW and MSM/TGW were conducted in the 3 cities Port Moresby, Lae, and Mount Hagen between June 2016 and December 2017. Survey eligibility criteria for FSW included: (1) born female, (2) >12 years of age, (3) able to speak English or Tok Pisin, and (4) had sold or exchanged sex with a man in the past 6 months. Survey eligibility for MSM/TGW included: (1) born male, (2) >12 years of age, (3) able to speak English or Tok Pisin, and (4) had engaged in oral or anal sex in the past 6 months with another person born male.

### Sample Size and Precision

We aimed to enroll 700 people into each BBS in each city. This assumed a design effect of 2 and was sufficiently powered to estimate an assumed HIV prevalence of 20% with an absolute precision of 10% [1]. The sample size was calculated so that the results of the present survey can be compared to anticipated follow-up studies (Multimedia Appendix 1, [13-15]).

### Unique Object Multiplier Method

Given the general unavailability of HIV testing or organization membership data in the survey cities, we primarily used the unique object multiplier (UOM) in conjunction with RDS surveys to estimate population size [16,17]. Volunteers, consisting of local KP peers and survey team members, distributed approximately 1000 keychains per population 1 week before survey rollout in each city. They were instructed to verify that each keychain recipient had not already received an object, that this person received only 1 object, and told the recipient to keep the object for the near future and not to give it to anyone else. Distributors were provided with 30 kina (US $12) for distributing keychains, irrespective of the actual number distributed—this removed any incentive to report distributing more keychains than actuality as may occur if the compensation was given per keychain distributed. They also received 5 kina (US $2) for transportation. To strengthen recall of receiving an object among BBS participants, keychain distributors wore distinctive hats that featured the survey logo “Kauntim mi tu.” During survey eligibility screening, the coupon manager asked participants if they received a unique object from a distributor. Those indicating that they had the unique object were asked to show it. If unable to do so, they were asked to select the keychain from among other keychains displayed by the coupon manager.

### Service Multiplier Method

The service multiplier method was used only in Port Moresby and Mount Hagen [10,18,19]. Survey participants were asked during the face-to-face interview whether they had tested for HIV at specific health facilities in 2015 (Port Moresby) or 2016 (Mount Hagen). Four HIV testing providers in Port Moresby and 1 in Mount Hagen were capable of providing key population-specific testing information. None were able to do so in Lae. Survey participants in each city were also asked whether they belonged to a KP organization. Responses to these questions were paired with data from HIV testing organizations and the KP community organization to develop multiple service multiplier estimates. In Lae and Mount Hagen, no KP organization could provide unique membership data.

### Successive Sampling Method: Population Size Estimation

Using Respondent-Driven Sampling Analyst (RDS-A) version 0.62 [20,21] the successive sampling-population size estimation (SS-PSE) method was used to produce size estimates using routinely collected data in RDS surveys including: (1) self-reported network size, (2) number of participant’s recruits enrolled in the survey, and (3) the date order of survey enrollment. We imputed visibility using these 3 routinely collected data items in order to smooth the network size distribution, reduce the effect of outliers, and minimize heaping of values (eg, around 5,10,15) [22]. Prior estimates were calculated using distribution of age and sex in each city, city general population sizes, proportions of FSW, MSM, and TGW in other countries, and previous knowledge of sex work in PNG.

### Data Analysis

Data were analyzed using RDS-A with the Gile SS-PSE and Microsoft Excel 2016. Standard formulas for the UOM method, service multiplier method, and SS-PSE were used [1]. Both weighted and unweighted estimates were produced for the multiplier methods to compare results. The 95% CI were calculated around point estimates using RDS-A.

### Protection of Minors

Participants <18 years were provided referrals as needed to organizations that offer counseling, protection, and other relevant services for victims of sexual exploitation and abuse.

### Ethical Approval

This survey was approved by the PNG National Department of Health’s Medical Research Advisory Committee, the Research Advisory Committee of the National AIDS Council Secretariat, the PNG Institute of Medical Research’s Institutional Review Board, and the Human Research Ethics Committee at University of New South Wales Sydney, Australia. The activity was reviewed according to the Centers for Disease Control and Prevention (CDC) human research protection procedures and was determined to be research but the CDC was not engaged in research collection. Two peer-led organizations for KP (ie, Friends Frangipani and Kapul Champions) provided letters of endorsement.

## Results

### Sampling and Recruitment

Among the FSW, 673 were enrolled in Port Moresby, 709 in Lae, and 709 in Mount Hagen while for MSM/TGW there were 400 enrolled in Port Moresby, 352 in Lae, and 111 in Mount Hagen (Table 1). Similarly, more keychains were distributed to FSW in Port Moresby, Lae, and Mount Hagen (N=867, N=790, N=546, respectively) compared to MSM/TGW (N=598, N=777, N=152, respectively), suggesting that FSW are easier to reach, and they are likely to be better networked. The number of keychains to be distributed was determined with a calculator found in international guidelines [1]; we aimed to distribute more keychains than our sample size. The RDS data collection took approximately the same amount of time for each city and population (14-20 weeks).

Tables 2 and 3 present population size estimates for KP in each city. For FSW (Table 2), population size estimates in Port Moresby ranged from 3537-35,048 (2.1%-20.7% of the adult female population), in Lae from 4482-6105 (10.5%-14.4% of the adult female population), and in Mount Hagen from 2386-6315 (15.5%-40.9% of the adult female population). For MSM/TGW (Table 3), population size estimates in Port Moresby ranged from 501-18,644 (0.3%-9.6% of the adult male population), in Lae from 3455-4669 (7.5%-10.1% of the adult male population), and in Mount Hagen from 1095-3625 (6.3%-20.8% of the adult male population).

**Table 1:** Description of surveys conducted among female sex workers (FSW) and men who have sex with men (MSM) and transgender women (TGW) in Papua New Guinea in 2016 and 2017.

Target population in each city		Enrolled participants, (N)	Keychains distributed, (N)	Keychains not distributed, (N)	Participants receiving keychains, (N)	Survey period	Data collection period, weeks
**FSW**							
	Port Moresby	673	867	133	51	Jun 2016-Nov 2016	20
	Lae	709	790	291	110	Jan 2017-May 2017	16
	Mount Hagen	709	546	454	71	Sep 2017-Dec 2017	14
**MSM/TGW**							
	Port Moresby	400	598	402	35	Jun 2016-Nov 2016	20
	Lae	352	777	223	75	Jan 2017-May 2017	17
	Mount Hagen	111	152	848	5	Sep 2017-Dec 2017	14

Port Moresby provided the most analytic possibilities, although there was variability in the number of unique individuals tested across these 4 organizations and only 1 KP organization could provide data on the number of members. As described above, in Lae, only the UOM and SS-PSE methods were used. In Mount Hagen, UOM, service multiplier (only for FSW), and SS-PSE estimates were derived for both KP. As many population estimates were developed in Port Moresby, meetings were held to discuss the estimates and the extent to which assumptions were met in order to identify a final estimate. In Lae and Mount Hagen, where fewer population estimates were developed, such meetings played a smaller role. Each of the 3 methods is direct and empirical, making them superior to other indirect methods such as census and enumeration [1]. These latter simplistic methods depend on counting everyone or a large number of people, which can be costly for census and often unfeasible for both with hidden groups like KP. Finally, weighted estimates were selected over unweighted estimates in order to increase the likelihood of independence between the convenient nature of keychain distribution (the capture) and RDS recruitment (the recapture) by turning the RDS sample data into population-based data. Without weighting of estimates, key assumptions of multiplier methods are violated.

### Population Size

Final weighted estimates chosen for each city were (1) 16,053 (95% CI 8232-23,874) FSW and 7487 (95% CI 3975-11,000) MSM/TGW in Port Moresby approximately 9.5% and 3.8% of the female and male populations, respectively, (2) 6105 (95% CI 4459-7752) FSW and 4669 (95% CI 3068-6271) MSM/TGW in Lae, approximately 14.4% and 10.1% of the female and male populations, respectively, and (3) 2646 (95% CI 1655-3638) FSW and 1095 (95% CI 913-1151) MSM/TGW in Mount Hagen approximately 17.1% and 6.3% of the female and male populations respectively (Tables 2 and 3)**.**

**Table 2:** Population size estimates for female sex workers (FSW) in Port Moresby, Lae, and Mount Hagen using unique object multiplier (UOM), service multipliers organizations (ORG1, ORG2, ORG3, ORG4), and successive sampling-population size estimation (SS-PSE) in 2015 and 2016.

Population size estimation method					Multiplier number, N		Survey, %		Estimate, 95% CI		Female urban population size based on total, %^a^
**Port Moresby, N=169,291^a^**											
	**UOM**										
		Unweighted			867		7.6		11,407 (8487-14,327)		6.7
		Weighted			867		5.4		16,053 (8232-23,874)		9.5
	**ORG1**										
		**KP membership**									
			Unweighted	3169		10.8		28,906 (22,764-35,048)		17.1	
			Weighted	3169		9.6		32,532 (30,324-34,743)		19.2	
		**HIV testing**									
			Unweighted	908		16.6		5464 (4599-6329)		3.2	
			Weighted	908		14.4		6328 (4383-8273)		3.7	
	**ORG2**										
		**HIV testing**									
			Unweighted	77		1.2		6487 (2257-10,717)		3.8	
			Weighted	77		1.9		3907 (432-7383)		2.3	
	**ORG3**										
		**HIV testing**									
			Unweighted	208		0.6		35,048 (1134-68,962)		20.7	
			Weighted	208		1		18,773 (0-39,354)		11.1	
	**ORG4**										
		**HIV testing**									
			Unweighted	63		0.4		14,154 (0-29,750)		8.4	
			Weighted	63		0.4		14,078 (0-31,388)		8.3	
	SS-PSE				—^b^		—^b^		3537 (1062-6870)		2.1
**Lae, N=42,532^a^**											
	**UOM**										
		Unweighted			790		15.5		5092 (4280-5903)		12
		Weighted			790		13		6105 (4459-7752)		14.4
	SS-PSE				—^b^		—^b^		4482 (1473-7388)		10.5
**Mount Hagen, N=15,430^a^**											
	**UOM**										
		Unweighted			546		10		5452 (4330-6574)		35.3
		Weighted			546		8.6		6315 (4668-7963)		40.9
	**ORG2**										
		**HIV testing**									
			Unweighted	138		5.8		2386 (1792-2981)		15.5	
			Weighted	138		5.2		2646 (1655-3638)		17.1	
	SS-PSE				—^b^		—^b^		3843 (1303-7989)		24.9

^a^Values provided by the 2011 census of Papua New Guinea.

^b^Not applicable.

**Table 3:** Population size estimates for men who have sex with men (MSM) and transgender women (TGW) in Port Moresby, Lae, and Mount Hagen using unique object multiplier (UOM), service multiplier organizations (ORG1, ORG2, ORG3, ORG4), and successive sampling-population size estimation (SS-PSE) in 2015 and 2016.

Population size estimation method				Multiplier number, N	Survey, %	Estimate, (95% CI)	Male urban population size based on total, %^a^
**Port Moresby, N=194,834^a^**							
	**UOM**						
		Unweighted		598	8.8	6834 (4735-8932)	3.5
		Weighted		598	8	7487 (3975-11,000)	3.8
	**ORG1**						
		**KP membership**					
			Unweighted	792	5.8	13,773 (8388-19,158)	7.1
			Weighted	792	4.2	18,644 (13,773-23,514)	9.6
		**HIV testing**					
			Unweighted	183	8	2288 (1597-2978)	1.2
			Weighted	183	7.8	2380 (1218-3543)	1.2
	**ORG2**						
		**HIV testing**					
			Unweighted	—^b^	—^b^	—^b^	—^b^
			Weighted	—^b^	—^b^	—^b^	—^b^
	**ORG3**						
		**HIV testing**					
			Unweighted	7	1.3	560 (299-821)	0.3
			Weighted	7	1.5	501 (0-1175)	0.3
	**ORG4**						
		**HIV testing**					
			Unweighted	8	0.8	1067 (116-2017)	0.5
			Weighted	8	0.3	2185 (0-4941)	1.1
	SS-PSE			—^c^	—^c^	3846 (3074-4200)	2
**Lae, N=46,076^a^**							
	**UOM**						
		Unweighted		777	21.3	3647 (2951-4343)	7.9
		Weighted		777	16.8	4669 (3068-6271)	10.1
	SS-PSE			—^c^	—^c^	3455 (2752-3672)	7.5
**Mount Hagen, N=17,400^a^**							
	**UOM**						
		Unweighted		152	4.5	3374 (532-6217)	19.4
		Weighted		152	4.2	3625 (754-6497)	20.8
	**ORG2**						
		**HIV testing**					
			Unweighted	—^b^	—^b^	—^b^	—^b^
			Weighted	—^b^	—^b^	—^b^	—^b^
	SS-PSE			—^c^	—^c^	1095 (913-1151)	6.3

^a^Values provided by the 2011 census of Papua New Guinea.

^b^Not available.

^c^Not applicable.

## Discussion

We employed several methods to develop population size estimates of FSW and MSM/TGW in PNG. We believe that the most robust population size estimates produced were with the UOM for both KP in Port Moresby and Lae, and in Mount Hagen, the service multiplier for FSW and SS-PSE for MSM/TGW. We present both unweighted and weighted estimates, the latter which adjusts for RDS recruitment. Individuals with smaller network sizes are up-weighted, whereby their responses are more valued as seen by the larger weighted estimates than unweighted estimates in our study.

Final population estimates were chosen through a series of rigorous meetings between experts in PNG, Australia, and the United States. Given the number and range of estimates produced, investigators reviewed each size estimation method, the extent to which their assumptions were met, and the resulting estimates. The investigators narrowed down the estimates to those that were most robust in each city. These estimates were then presented to key stakeholders including the National Department of Health, key population organizations, donors, and United Nation agencies who were tasked with agreeing on a single estimate. Details and results of these discussions follow below.

Several precautionary steps were taken to maximize the utility and accuracy of the unique object multiplier. We minimized the risk of participants falsely indicating that they had received a keychain when in fact they had not by asking participants to (1) show their keychain, or (2) identify the correct keychain from a group consisting of this keychain and other incorrect keychains. Distributing the unique object approximately one week before the start of the RDS survey helped to (1) limit in and out-migration of participants, (2) minimize the possibility of target population members giving their object away, and (3) reduce the possibility of someone receiving objects from multiple distributors.

It is worth noting that fewer keychains were distributed to MSM/TGW, which may be explained by their lower propensity toward gathering in public places due to stigma and discrimination and smaller network sizes, as compared to FSW. While keychain distribution is influenced partially by how well a distributor is trained, smaller network sizes pose a challenge because fewer people may be present in KP-friendly hotspots and there is greater reliance on tapping into an individual’s limited social connections to distribute these keychains. We tried to address stigma/discrimination barriers in Mount Hagen prior to study initiation by building trust and rapport. For future work, we would consider using more volunteers who each distribute fewer objects, since individuals may know fewer people due to stigma.

We encountered no problems distributing keychains to either population and leftover keychains were returned to survey sites, suggesting that keychain distribution occurred with fidelity. In Mount Hagen, UOM estimates indicated that FSW account for approximately 40% of the adult female population and MSM/TGW 20% of the adult male population. (Tables 2 and 3). These estimates were deemed unreasonably high, leading to their exclusion in favor of the service multiplier and SS-PSE estimates, respectively, in this city. All MSM/TGW estimates in Mount Hagen were hampered by the small RDS sample recruited.

While the data provided by HIV testing organizations in Port Moresby were meant to represent unique individuals, some service multiplier results there were excluded due to very wide CI values, while other population size estimates were too low to be plausible given our understanding of sex work in this city, and MSM/TGW globally [16]. Our results are higher than results from a previous survey in Port Moresby [10], which reported FSW and MSM service multiplier estimates to be 4212 (95% CI 3586-4839) and 2126 (1787-2468), respectively. We believe that our results are acceptable because those population estimates are almost a decade old, Port Moresby has undergone tremendous growth since then, and we included transgender women in our estimates.

Given the age of previous PNG estimates, we lacked a recent and more accurate prior value for use in producing an estimate with the SS-PSE method, suggesting a reason why the results from this method were relatively low. Likewise, the SS-PSE method estimates the number of people that fit our survey’s time-frame eligibility criteria (ie, behaviors in the past 6 months) [23]. Therefore, individuals who sold sex, engaged in same-sex sexual behaviors, or TGW who had sex with men more than six months ago are not included in this estimate. As these people are still KP and face multiple vulnerabilities, we felt it important to select a larger and more plausible estimate, hence the selection of the UOM for Port Moresby and Lae. In both cities, the UOM produced population size estimates that were slightly larger than SS-PSE.

Our findings are limited in several ways, beginning with the self-reported nature of the interview data. Participants may have chosen to underreport or overreport HIV testing or organization membership due to stigma or fear, resulting in overestimation and underestimation, respectively. This social desirability bias could be mitigated in the future through the use of computer-assisted self-interviews [24-26].

The unique object multiplier relies on accurate reporting of keychains given out by distributors and received by KP. We cannot know whether all keychains that were said to be distributed were actually distributed correctly, that individuals received only one keychain each, or how many keychain recipients were ineligible for the survey. Additionally, though we distributed keychains immediately before survey initiation, data collection took up to four months to complete, so it is possible that KP members migrated out of the catchment area, causing an underestimation.

Few HIV testing providers in Port Moresby and Mount Hagen were able to provide data and those that were able tested only a small number of KP. HIV testing data were nonexistent in Lae, meaning the service multiplier method could not be used. There is also the concern of recall bias for participants in Port Moresby who were asked about services accessed in 2015. Furthermore, SS-PSE relies on prior estimates, which themselves may not be accurate in Port Moresby and we did not have in either Lae or Mount Hagen.

Our findings are important for describing the number of FSW and MSM/TGW in the survey cities. The direct and empirical size estimation methods used in this survey are superior to other indirect methods, such as census and enumeration. The population size estimates in this survey will inform efforts to improve resource targeting and monitoring of both existing and new services for these key populations. HIV testing providers in PNG should be encouraged to disaggregate their data by key population to facilitate the use of the service multiplier. This will also increase the utility of routinely collected data.

### Lessons Learned

Much of this project’s success, which was led by the national government of PNG with technical assistance from the United States and Australia, should be credited to the Papua New Guinean “Kaumtim mi tu” survey team, which included KP members in strategic staff positions, and who nurtured trust among KP in each city. The staff was crucial in organizing keychain distribution and obtaining service provider data. In addition, the survey team was almost unchanged across all cities. This resulted in a consistency of operations and data collection, improved efficiency, and an overall increase in technical capacity to implement RDS surveys and population size estimation activities. We also used tablets to collect data electronically, which simplified data management and decreased chances of error. Furthermore, we found it valuable to start our project with the “easiest” site first, Port Moresby, because the capital city had the most visible KP, facilitating KP engagement and survey implementation. News of the survey’s benefits to individuals and KP as a whole, as well as the friendliness and professionalism of the survey team, traveled to Lae and Mount Hagen motivating participation there. Given the lack of recent population size estimates, we found it indispensable to use several methods to estimate population size because each method has its strengths and weaknesses.

## Appendix

Multimedia Appendix 1 Study sample size and precision calculations.

## Abbreviations

BBS: biobehavioral surveys
CDC: Centers for Disease Control and Prevention
FSW: female sex workers
KP: key populations
MSM: men who have sex with men
ORG: organizations
PNG: Papua New Guinea
RDS: respondent-driven sampling
SS-PSE: successive sampling-population size estimation
TGW: transgender women
UOM: unique object multiplier
