# Supplementary material for: Kuantim mi tu (“Count me too”): Using Multiple Methods to Estimate the Number of Female Sex Workers, Men Who Have Sex With Men, and Transgender Women in Papua New Guinea in 2016 and 2017
Source: JMIR Public Health Surveill. 2019 Mar 21;5(1):e11285. doi: 10.2196/11285 (PMC6447989; doi:10.2196/11285)
Supplement: Multimedia Appendix 1 [file publichealth_v5i1e11285_app1.pdf]

Multimedia Appendix 1: Sample Size and Precision Calculations

Given that this data was collected as part of a large-scale IBBS, our goal was to ensure that presently collected data could be compared to anticipated studies in the near future. Thus, the formula used to calculate sample size was one that compares two proportions:

$$n = \frac{D\left[Z_{1-\alpha}\sqrt{2\bar{P}(1-\bar{P})} + Z_{1-\beta}\sqrt{P_1(1-P_1)+P_2(1-P_2)}\right]^2}{(P_2-P_1)^2}$$

where *D* is the design effect, *P1* is the estimated proportion at the time of the first survey, *P2* is the proportion at some future date (such that the quantity *(P2 - P1)* is the size of the magnitude of change that one wishes to be able to reliably detect), *P* is equal to (*P1 + P2) / 2*, Z_1 *- a* is the z-score corresponding to the probability with which one can be certain that an observed change of size *(P2 - P1)* did not occur by chance (that is, the level of significance), and Z_1 - *ß* is the z-score corresponding to the probability with which one wishes to be certain that a change of size *(P2 - P1)* will be detected (that is, the power of the survey). The HIV prevalence among the general population in PNG has remained under 1% for over five years. [11] HIV prevalence among sex workers (male and female combined) is estimated at 16.9% in Port Moresby, NCD [24] and 2.7% for FSW in Eastern Highlands [25], and is unknown among MSM. [26] We thus proposed using an estimated HIV prevalence of 20% and an absolute precision of 10%. A design effect of two was selected, as this adheres to RDS standards and assumptions. [1] Therefore, the effective sample size is estimated to be 350 participants and the target sample size is 700 individuals when accounting for design effect. The confidence intervals reflect that of a random sample with 350 participants.
